# A perspective on the environmental impact of plant-based protein concentrates and isolates

**DOI:** 10.1073/pnas.2319003121

**Published:** 2024-12-02

**Authors:** William R. Aimutis, Rohan Shirwaiker

**Affiliations:** ^a^North Carolina Food Innovation Lab, North Carolina State University, Kannapolis, NC 28081; ^b^Bezos Center for Sustainable Protein at NC State, North Carolina State University, Raleigh, NC 27606; ^c^Edward P. Fitts Department of Industrial and Systems Engineering, North Carolina State University, Raleigh, NC 27695

**Keywords:** plant-based, protein, environmental impact, food security

## Abstract

Plant-based proteins are promoted as nutritious, environmentally safe, and a key element for food security. Numerous studies using environmental life cycle assessments confirmed plant-based meat alternatives are more environmentally favorable than animal-derived meat. However, most missed other factors from protein extraction and concentration that contribute to environmental impact (both positive and negative). Furthermore, few studies examined the entire environmental impact because they overlooked social and nutritional life cycle assessments. Many publications assumed most environmental impact occurred in crop cultivation before high protein products are manufactured. However, industrial plant-based protein extraction, concentration, and purification require large amounts of natural resources, energy, chemicals, and highly specialized equipment seldom accurately assessed from an environmental, social, and nutritional perspective. This paper presents opinions and perspectives on additional information to consider to fully realize environmental benefits of highly proteinaceous plant-based food ingredients. Elements are also discussed we perceive as “hot spots” for future assessments in presenting a complete picture for minimizing environmental impact and how the numerous actors in this supply chain can transform our food system and secure protein availability for future generations.

Livestock production has a significant environmental and climate footprint. Meat and dairy protein production accounts for approximately 20% of the global food energy but utilizes 70% of agricultural land, 40% of arable cropland, and generates over 70% of food-related greenhouse gas emissions (GHGe) (1–4). Additionally, their freshwater footprint exceeds 25%. There are other negative consequences of intensive livestock production including biodiversity losses in some regions, water eutrophication from manure and urine runoff, and soil acidification.

Traditionally, consumers favor protein from meat, eggs, and dairy products, but consumers are purchasing more foods enriched with plant-based protein (PBP) (5). Advocates for PBP promote that they are 1) better for our environment than animal-derived proteins (ADP), 2) healthier food options, and 3) ethically superior because less animals are slaughtered. Common conjecture cites these products will mitigate climate change with a lower carbon footprint compared to ADP.

PBP are traditionally extracted from ground meals remaining after oil and starches are extracted from grains and cereals as food ingredients. The meal is further processed to manufacture concentrates (PBPC; protein content <85%) and isolates (PBPI; protein content >85%). Many plant-based (PB) foods are formulated with PBPC and PBPI, especially with proteins concentrated from soy and wheat. Recently, consumers are expressing concerns about soy and wheat being allergenic and having off-flavors resulting in increased offerings from suppliers of yellow pea, rice, lentils, chickpea, and other pulse proteins, in the form of concentrates and isolates. Additionally, PBP manufacturers are extracting proteins from upcycled products (e.g., spent brewers’ and distillers’ grains) to produce lower-cost PBPC and PBPI.

The Plant Based Foods Association (6) reports sales growth of 45% ($8 billion in total sales) from 2019 to 2022 across multiple food categories. PBP in the United States are increasingly used in multiple food formulations, especially “meat” and “dairy” alternatives. Plant-based dairy alternatives (PBDA) were the first products marketed to consumers despite being nutritionally inferior to bovine milk. Plant-based meat alternatives (PBMA) formulated with PBPC and/or PBPI quickly followed and emerged as an improved protein source though essential amino acid content might be lacking.

Several reasons are emerging to promote consumption of sustainable PBP. An important reason is rapid population growth in coming decades will place a burden on our entire food supply chain to meet nutritional and food security needs. The global protein supply chain will require 40% additional protein by 2050 as a global population consumes 67% more meat (7). Increased protein supply could be accomplished by increasing livestock numbers, but environmental impact will be potentially tragic to planet Earth because of GHGe, water and land pollution, and negative consequences to biodiversity. If appropriate mitigation strategies including adoption of sustainable food technologies are not enacted soon, increased demand on our food systems is projected to increase adverse environmental impacts 50 to 90% by 2050 when the global population is estimated to be 9.8 billion people (8, 9).

Several publications show that PB food products are environmentally better (10–12). Many advocates now equate all PBP products as better than ADP. However, in most studies, a critical issue overlooked or minimized is these products are formulated with appreciable amounts of PBPC and/or PBPI. Very few studies examine the entire environmental impact in producing concentrates and isolates. Many publications assume that most environmental impact occurs in crop cultivation before PBPC and PBPI are manufactured. However, industrial PBP extraction, concentration, and purification require large amounts of natural resources, energy, chemicals, and highly specialized equipment seldom accurately assessed from an environmental, social, and nutritional perspective. This paper presents opinions and perspectives on other information to consider when analyzing environmental impact of PBPC and PBPI. We first examine trends in global protein consumption, emphasizing the importance of sustainable dietary choices for health and environment. We then discuss the evolution and current state of PBP products, highlighting key manufacturing processes, environmental considerations, and industry challenges. Finally, we summarize traditional environmental sustainability assessments and discuss what we perceive as “hot spots” for consideration in presenting a complete picture of PBP environmental impact.

## Balancing Nutritional Security and Environmental Sustainability

Proteins are essential dietary nutrients for humans from animal and plant sources. Meat is the preferred protein source in the human diet (Table 1). A majority (>80%) of the global population are meat consumers because of cultural factors, gender, geographical location, availability, and affordability (13). Per capita protein (regardless of form—animal or plant) consumption is greatest in (rank order) Iceland, Hong Kong, Lithuania, Israel, and Albania (1). The United States was ninth. The United States, Argentina, Australia, Brazil, Spain, and Mongolia consume the most meat. These six countries alone are eating 30 to 55% more protein than the daily recommended amounts (50 g of protein/day) needed for good nutrition. In recent years, per capita protein consumption increased as societies become more affluent. For example, Angola, Afghanistan, Solomon Islands, Yemen, and Uganda are increasing their annual per capita protein consumption (1). Other countries with rapidly growing populations like Ethiopia, Botswana, Niger, Namibia, Somalia, Mali, and Zimbabwe are also eating more protein from various sources including meat. As our global population and protein consumption increases, there is a need to globally reduce protein intake to sustain future human life in a healthy, nutritional manner. This is achievable in balance with animal agriculture by diversifying our diets with multiple protein sources, diverting edible protein consumed by other species (for example, companion animals and aquaculture) to less desirable protein sources (e.g., insect and algae), and further developing a sustainable protein supply chain using technology to increase and improve PBP extraction and concentration production methods.

**Table 1.** Global production and consumption of animal-derived proteins^*^

Protein source	Product average protein content (%)	Total global production of source raw material (M mT)	Global amount of consumable protein (M mT)	Leading per capita consuming continents^†^	Rapidly growing per capita consuming continents^†^
Meat	26	880	229	NA, EU, SA, AX	AF, AS
Milk	3.5	897	31.4	AS (India), EU, NA	AS, AF
Eggs	12.0	87	10.4	AS (Japan), NA, SA	AS (China), SA, NA, EU
Fish/Shellfish	26.0	185	48	AS, EU	AS Pacific

^*^Reference: (1).

^†^Abbreviations: AF, Africa; AX, Australia; AS, Asia; EU, Europe; NA, North America; SA, South America.

A high-quality protein, regardless of source, must support human growth and development while being readily digested and absorbed (14). Protein digestibility-corrected amino acid scores (PDCAAS) were originally used to quantify protein’s nutritional quality, but to overcome test limitations nutritionists now use the digestible indispensable amino acid scores (DIASS) to demonstrate equivalency more accurately to ADP (15). This method assigns scores considering the limiting indispensable amino acids (IAAs) within a given food (or meal) (16). Protein varieties vary in their IAA contents which impacts digestibility (Table 2).

**Table 2.** Digestibility indices of proteins commonly used in food formulations

Protein source	PDCAAS^*^	DIAAS^*^	References
Animal			
Cow milk	1.00	1.16	(17)
Eggs	1.00	1.16	(17)
Beef	1.00	1.12	(17)
Poultry	1.00	1.08	(17)
Pork	1.00	1.14	(17)
Seafood	1.00	–	(18)
Plant			
Oat	0.57	0.77	(19)
Soy	1.00	0.99	(17)
Rice	–	0.64	(17)
Wheat	0.45	0.40	(17)
Chickpea	0.84	0.82	(20)
Pea	0.78	1.00	(17)
Fava beans	0.58	0.54	(21)
Green lentils	0.91	0.49	(22)
Lupin	–	0.68	(23)
Hemp	–	0.54	(23)
Quinoa	0.79	--	(18)

^*^Abbreviations: PDCAAS, Protein Digestibility-Corrected Amino Acid Score; DIAAS, Digestible Indispensable Amino Acid Score.

Many PBP lack appreciable levels of lysine, valine, branched-chain, and sulfur-containing methionine and cysteine amino acids (except for grain-sourced proteins). Nutritional critics believe PBP are less nutritionally complete than ADP because of these amino acid deficiencies (4). However, a recent study reported that these parameters do not correlate well with growth performance data (3). When adequate amounts of sole-sourced PBP are used in a formulation, they performed equivalent to casein (considered a gold reference standard) in longitudinal bone growth studies. There is also concern PBP may not support muscle anabolism to prevent skeletal muscle loss as aging progresses. A study with older consumers reported when an ample protein amount is consumed skeletal muscle remodeling is facilitated (4). However, there are a limited number of studies in both areas. Fortunately, most of us consume proteins from multiple sources and our daily requirements for all our essential amino acids will likely be met.

Other potential nutritional issues face consumers shifting diets from ADP domination to PBMA and PBDA. Consumers in developed countries consume an adequate balance of PBP to meet protein nutritional needs as they reduce ADP intake, but consumers in emerging countries should be aware of their protein quantity and quality intake (24, 25). Many PBMA and PBDA utilize proteins with lower nutritional quality than traditional meat and dairy (Table 2). Manufacturers of PBP products have offered consumers a narrow variety of formats (burgers, nuggets, meatballs, sausages, sweetened beverages, etc.) with very diverse formulations and nutritional composition to replace traditional meat and dairy products. Unfortunately, these product offerings, even though PB, are often less nutritious than minimally processed beans and legumes typical of vegetarian and vegan diets. To improve flavor and texture, food formulators have inadvertently developed products lower in protein and higher in sodium, fat, and sugar than nutritionists recommend. Consequently, PB-formulated foods are often deficient in calcium, potassium, magnesium, zinc, and Vitamin B12 (26). As more consumers become aware of these insufficiencies, they are refining their diets to being “flexitarians” eating meat and dairy in balance with PB offerings viewing this as healthier while helping the environment and animal welfare.

A majority of global GHGe are created at the primary production stage (farm-level crop and animal production), and less than 10% are produced in food manufacturing establishments (27, 28). All food supply chain actors are impacted by cries for food system transformation including those processing agricultural commodities and by-products to market protein concentrates and isolates. The food industry is facing multiple challenges, especially climate change and food security, as global population increases. Traditional triple bottom line factors of time, quality, and flexibility demand focus from operations personnel (29–31), but factors related to sustainability are rapidly increasing obligations resulting in a unique definition for the triple bottom line of people, planet, and profit (32, 33). Suddenly food product developers are designing products not only meeting consumer’s demands of tasting good, being convenient, and reasonably priced to additionally utilizing ingredients mitigating anthropogenic climate change to meet future health and sustainability goals. However, if industry must reach a future goal where 100% of products are recyclable and manufacturing yields zero environmental impact, the burden cannot only be on product developers and industry. Consumers must significantly adjust their current diets by consuming reasonable protein amounts and increase receptiveness to novel or future food choices through protein alternatives including insect, mycoprotein, and microalgae as options to replace animal-sourced foods.

## Plant-Based Protein and Food Formulation

Soy-based tofu, tempeh, and seitan are the simplest protein formats to produce meat-alternative products. These PBP were produced and consumed for many years in Asian countries, but in the United States, soy protein concentrate (SPC) originated in the late 1950s with little information about their nutritional properties and physical functionality (foaming, gelling, emulsifying, gelling, etc.). Early PBP products were combined with ground meat and marketed at reduced cost compared to traditional formats. Western consumers were not as accepting of differences in flavor, texture, and structure caused by soy inclusion, and category growth was stymied. Food engineers at General Mills, Inc. in 1964 began extruding SPC along with smoke and meat flavors to offer consumers a product resembling bacon crumbles (BaCos™). At the time it was considered a “Food of the Future” and it is now believed this was the origination of low moisture extruded PBMA currently marketed (34). Similarly, soy-based beverages were developed in the same era for consumers having dairy allergies. These products maintained static growth for several decades until recent demand for PBDA and PBMA began increasing. Several soy protein manufacturers have persevered over 50 y by developing new SPC and soy protein isolates (SPI) with multiple physical functionalities. These same manufacturers conducted nutritional studies demonstrating physiological effects from their products including regulatory approval that soy protein as part of a healthy diet could lower cholesterol and provide other cardiovascular benefits although this was repeatedly disputed based on lack of solid scientific evidence. Other PBP sources have slowly emerged in the last twenty years especially those sourced from yellow peas and wheat as industry and consumers became concerned about GMOs, soy protein allergenicity, and deforestation impact in heavily soy-cultivated regions. The PBP portfolio has expanded as agronomists and scientists characterized proteins from different raw materials including fava beans, chickpeas, rice, and other botanicals and their by-products for use in PB foods alone or combined with SPC or SPI (35).

**Fig. 1.**
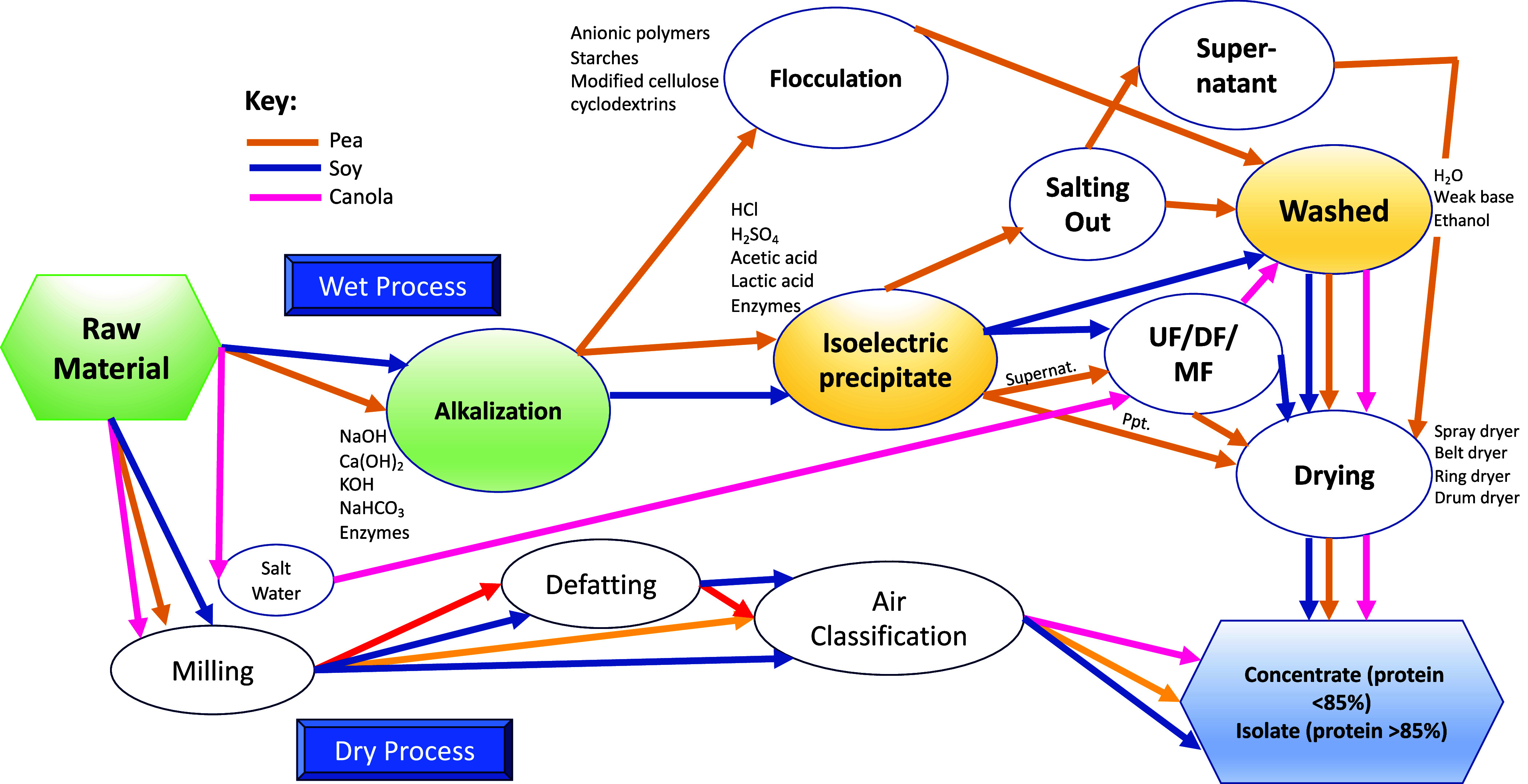
Complexity of PBP manufacturing from a variety for raw materials. This process flow accounts for the steps of extraction, concentration, purification, and drying. Although most commercial PBP manufacturers utilize proprietary processes, they all follow steps like those in this diagram.

Four unit operations are used in PBPC and PBPI manufacture—extraction, concentration, further purification (isolates only), and drying. However, this is oversimplifying a complicated manufacturing regime with multiple steps and processes impacting the environment (Fig. 1). Proteins are extracted from plant matrices using dry or wet methods (36). Selection of suitable protein extraction methods is dependent on the raw biomass source because pectin, cellulose, and hemicellulose fibers entrap protein molecules in their molecular structure. Biomass naturally low in residual fat (e.g., peas and other legumes, fava bean, hemp seed, quinoa, and others) or that can be defatted after initial processing steps is suitable for dry extraction methods using air classification or electrostatic separation. While this method is an energy-efficient process, it does use large amounts of electricity to power the mills and air cyclones needed for separation at scale.

Major oilseed crops (soy, canola, sunflower, and safflower) are traditionally extracted with hexane leaving a meal containing residual oils. To be dry processed, this material must be further extracted with environmentally friendly supercritical carbon dioxide. This unscaled process is a drawback to this extraction method, and therefore, these crops are further subjected to wet extraction methods. Conventional PBP wet extraction techniques use alkalization and acidification processes in their unit operations. These methods are only efficient at removing approximately 50% of the protein present in plant biomass and use harsh acidic and alkaline solvents for extraction (37). Alternatively, some manufacturers further solubilize proteins from initial extraction stages using conventional protein solubilizing by “salting out” molecules from an alkalized solution. This method uses appreciable amounts of sodium chloride that must be removed downstream when wastewater is further treated before release to the environment or municipalities (37). Enzymes are also frequently used to degrade cell walls and membranes to release proteins from plant matrices. This technique is especially useful in upcycling of brewers’ and distillers’ spent grains (38). Several good reviews on PBPC/PBPI processing were recently published (35–39).

Most PBMA products are manufactured by combining PBPC/PBPI (60 to 70% of the formula) with fibers, flavors, oil, and other minor ingredients followed by twin-screw extrusion and cooking. Extrusion utilizes thermal energy to align protein molecules in a finished product texture resembling ADP meats.

The recent surge in PBP growth transpired when dairy protein costs and supply chain availability issues occurred from 2011 to 2014 specifically for high-protein concentrates and isolates (40). In retaliation, frustrated food formulators began seeking alternatives and developed new formulas based on SPC, SPI, and other emerging PBPC and PBPI. Coincidently, the PB food movement was building momentum, and many new PBP product offerings were being launched (6). Several large food ingredient manufacturers in the starch and fiber sectors concurrently recognized several production “waste” side streams enriched in protein content could be valorized. Advantageously, using a valorized side-stream is a lower carbon footprint because primary products (starch, fiber, etc.) are mostly responsible for environmental burden (41). The quickest path to market was to utilize existing protein manufacturing facilities originally designed for soy or dairy proteins with little regard to environmental sustainability especially energy (natural gas and electric) and water usage.

The largest volume categories PBP will positively impact our environment and ultimately climate change are PBMA and PBDA. Environmental assessments have addressed PBPC/PBPI as agricultural commodities whose environmental impact is accounted mostly in the farm field. However, there are several nuances potentially adverse to our environment. Environmental assessments comparing PBMA to beef unequivocally confirm an impact on reducing multiple environmental markers. However, pork and poultry PBMA are only marginally better, and the gap of environmental friendliness may be narrowed if protein processing and extrusion were accounted for in environmental assessments. To accurately judge PBMA and PBDA environmental impact requires assessment of PBPC and PCPI manufacturing processes including all steps involved in extraction, concentration, purification, and drying (Fig. 1). This analysis is complex with numerous boundaries and chemical inputs integrated in the process. Unfortunately, PBPC and PBPI manufacturers often use different methods and process controls considered to be trade secrets making data comparison difficult.

Popularity of PBP products has ebbed and flowed the past five years. Consumers more readily accepted PBDA beverages than PBMA and egg products. Several different PBMA formats were marketed with mixed consumer reaction. While consumers were initially willing to partake in the interest of environmental sustainability, once they tried PB burgers or “chicken” nuggets/strips, they found themselves gravitating back to traditional meat products for flavors and textures. Additionally, PBMA ingredient labeling complexity and excessiveness were questioned by many consumers. Most consumers favor taste, price, healthy nutrition, clean label, and convenience as their priorities when choosing food products.

## Traditional Environmental Sustainability Assessments

Many governments, activist groups, and popular media are vilifying the agri-food industry as directly responsible for climate change and insinuating these negative aspects will ultimately impact global food security (42). This current crisis cannot be simply technology mitigated but will require actions that are institutional, financial, and behavioral (43). Sustainable Development Goals (SDG) established by the United Nations as strategic priorities initially focused on low-carbon agriculture, sustainable healthy diets, integrated biomass and bioenergy solutions, and food loss and waste reduction (42). This necessitated the incorporation of sustainability goals into core business operations and corporate sustainability plans (44). The food sector is complex with multiple actors including farmers, food manufacturers, retailers, suppliers, and ultimately the consumer; therefore, collaborations and partnerships must be mustered within the ecosystem to achieve these goals (45).

The sustainability concept implies civilization to be cognizant and diligent in using resources and products in a manner to preserve the quality of life for future generations (31, 46). Several data inputs are important for analyzing sustainability of proteinaceous food ingredients and finished foods including raw material agricultural practices, supply chain requirements, production processes, product use, distribution, storage conditions, and shelf life of both raw materials and finished foods (31). Energy and water use, waste, and factory emissions are input boundaries contributing to environmental friendliness of a sustainable manufacturing process. Several other process components are ignored including impact from by-products, heavy sanitation chemical use, protein extraction and concentration processes, equipment sterilization, and regulatory required equipment composition (stainless steel).

Multiple assessments have correlated technical, environmental, social, and nutritional information of protein products and processes impact on sustainability and climate (Table 3). While these models are accepted as standard practices, they are often limited in scope and data reliability because usually their data are specific to a particular manufacturing process including raw material sourcing and specifications, processing equipment design and scale, waste and by-product generation and treatment, and final product specifications. The inclusion of a TEA allows comparisons between studies. In some instances, information is unavailable while designing a process or is provided by preproduction runs from pilot plant equipment with potentially an inferior correlation to mass production. ISO standards have organized and codified factors to correlate data between parties for comparisons (47). The established critical factors declare a LCA’s intended purpose by explicitly defining goal and scope including boundaries being analyzed (for example “cradle to grave”). However, ISO has not clearly defined standardized data reporting (47). Furthermore, these methods are industry agnostic and as such are applied to a large variety of processes and applications.

**Table 3.** Assessment models typically used to study environmental impact of processes and products

Model	Purpose	Input (Functional units, FU)	Output	References
Life cycle costing (LCC)	Economic sustainability of a new facility or process	Multiple financials including initial capital, ongoing service, preventative maintenance, ongoing operations, and facility disposal (decommissioning)	Economic viability of a new plant, process, or product	(48–50)
Techno-economic analysis (TEA)	Assess technical and financial aspects of new products and processes	Process flow chart detailed with energy and mass flow at every step	Production costs; energy usage; by-product generation and upcycle use; predicts return on investment	(51, 52)
Environmental life cycle assessment (eLCA)	Quantify environmental impacts over a process or product life cycle	Environmental footprint at every process step of a product/process from beginning to end including usage of energy, natural resources, chemicals, packaging	Quantified environmental footprints from cradle to grave including GHGe, natural resource usage, potential water and air pollution, potential impact to global warming	(47–54)
Social life cycle analysis (sLCA)	Evaluates social and socioeconomic aspects of a product	Wages, working conditions, exposure to hazards,	Potential social impact of a process or product; data are more qualitative	(55, 56)
Nutritional life cycle analysis (nLCA)	Identifies protein quality in relation to the environmental expense of producing	Protein concentration, amino acid balance, protein bioavailability and digestibility, and micronutrient composition	Defines the environmental cost of producing highly refined protein products	(15, 57–59)

Transparency by commercial entities is critical for acceptance of these assessment methods by the public and regulatory agencies setting environmental policy. Recently, several food companies were exposed by environmental watchdog groups of greenwashing their environmental data making themselves appear more sustainable (60, 61). Disinformation undoubtedly contributes to more media hyperbole to use against PBP and its affiliated products industry.

A significant volume of PBP research and PBMA/PBDA product development has led to several reviews being published the last few years (62–66). An increasing volume of publications review PBP product sustainability, and the consensus is that these products are environmentally good with the potential to mitigate climate change (12, 35, 67–73). Fewer publications (including those cited above) reported the environmental footprint of only PBPC and PBPI (51, 73–91). In many cases, the published data are difficult to correlate as mentioned earlier. Nevertheless, there are top-line interpretations. Land use of common PBP crops was less than animal agriculture (Table 4). There was consensus in the cultivation stage fertilizers, fuel, and crop yield have a significant climate impact. Cultivation locations and methods influenced environmental impact partially because of different climates affecting crop yield or distance to a processing facility influencing fuel usage. In PBP manufacturing, extraction and drying stages are the major source of GHGe. Similarly, further PBP refinement or purification (Table 4) negatively influences climate impact by greater energy usage especially for cereal crops (78). Very few studies examined environmental sustainability using LCC, TEA, or sLCA, challenging the ability to accurately model or compare various systems and their process stages. Furthermore, many PBP processes were based on laboratory extraction and concentration, or processes used for other proteins with an assumption data input would be similar.

**Table 4.** Environmental impact of plant-based crops and proteins used for alternative meat and dairy products^*^^,^^†^^,^^‡^

Crop	Environmental metric	Cultivation impact	Protein concentrate	Protein isolate
Legumes (soy, peas, and lupin)	GHGe (kg CO_2_ eq.)	0.2 to 0.6	0.7 to 2.0	1.8 to 1.3
	LU (m^2^ annual)	3.0	8.0 to 20.8	5.8 to 34.7
Cereals (wheat, oats)	GHGe (kg CO_2_ eq.)	0.3 to 1.0	3.3	2.1 to 8.8
	LU (m^2^ annual)	2.7 to 5.5	3.2	8.6 to 33.5

^*^Abbreviations: GHGe, greenhouse gas emission; LU, land use.

^†^Values are per Kg processed crop.

^‡^References: (78, 89, 92).

Consumers and analysts cite several factors important to future PBP acceptance, and ultimately commercial success by the public. These factors include environmentally friendly, safe for both manufacturers and consumers, nutritional equivalence to ADP, and cost parity or less expensive. To unequivocally convince naysayers that PBP are environmentally comparable or better than traditional animal proteins will minimally require the development of databases on environmental impacts associated with protein extraction, concentration, isolation, and drying. In a perfect world, the simplest protein extraction would be accomplished using hot water extraction, but PBPC are extracted using either milling followed by air classification (91–94) or more hostile extraction procedures mentioned earlier. Processing of PBP is water (hydration and rinsing steps) and energy (agitation) intensive, acids and bases are not environmentally friendly, and spray drying requires natural gas and air blowers. Processing unit operations and conditions are typically unique to individual PBP manufacturers usually with an unwillingness to share outside their respective organization. Many of the published PB food assessments neglect processing impact in their analysis, or they only superficially cover the subject from a processing aid cost for acids and bases.

The PBP industry is commercializing information from eLCA studies despite practitioners’ concerns about incomplete and unreliable data sources with little codification of assessment methods. Credibility of eLCA will only be accepted when reliable levels of information completeness are available in a format easily understood by the public. For acceptance, multiple PBP products manufactured by multiple methods should be assessed. Ultimately, analysis methods must be reviewed and aligned to identify necessary data points to compare environmental impact of a growing PBP supply chain manufactured in several different geographical areas at production scale. As this occurs, verification and validation of the analysis models with real operational data will be key to wider acceptance and adoption.

## Future Environmental Assessment Elements

Many eLCAs ignore extensive stainless-steel usage in biorefinery environments. Stainless steel is manufactured from Fe, Cr, Ni, and Mb ores in a very thermal-intensive process combining the minerals and ultimately casting or fabricating final products (pipes, reactors, etc.). This environmentally impactful process contributes to global warming, extensive water usage and pollution, air pollution, biodiversity destruction, and extensive land use for mining and producing stainless steel (95). While many tons of PBP can be manufactured by continual equipment reutilization, it is still considered a hotspot not clearly defined from an environmental impact perspective. Traditional ADP processing plants also use large amounts of stainless, but not as extensive. The cleaning, sanitizing, and occasionally sterilizing of large plants dominated by stainless steel is an environmental factor seldom discussed when analyzing ADP or PBP manufacture in an eLCA.

Engineers and environmental critics are eager to identify stainless steel alternatives, and a greater need exists with emergence of protein products in all three manufacturing domains—traditional PB extraction and concentration, precision fermentation, and cell cultivation. Metallurgists are proposing metal matrix composites (MMC) to reduce future needs for stainless (96). MMC are manufactured by embedding other metals, ceramics, or organic compounds as reinforcing materials into stainless steel during the manufacturing process with a resultant material less reliant on minerals used to manufacture stainless steel. This ultimately reduces the environmental impact of mining Ni, Cr, and Mb. This technology is in development but being extensively tested. Laboratory studies indicate that MMC resists corrosion and wear like stainless steel. Repurposing and upfitting stainless steel equipment from underutilized or stranded biomass processing and pharmaceutical facilities toward PBP manufacturing has also been an ongoing discussion addressing this challenge, although formal environmental and economic assessments are lacking (97, 98).

Some PBP are isolated as coproducts from other processes manufacturing starches, fibers, or other value-added products. The few published studies under this scenario report improved environmental sustainability factors because many production costs and environmental impact factors occur prior to protein extraction and concentration (51, 99). Furthermore, improved databases are needed with products repeatedly produced at commercial scale. Many PBP manufacturers have proprietary process data including the solids percentage being used for initial extraction. These factors ultimately affect PBPC and PBPI market prices and commercial use of these products. Increased product demand above manufacturing levels quantified in an eLCA could negatively influence environmental factors while simultaneously improving production efficiency. Clearly, this scenario needs study with extensive sensitivity analysis to fully comprehend environmental impact. Further robustness of these models will provide more accurate and precise eLCA and provide validated information for continuous improvement in the manufacturing theatre (100).

PBP manufacturing technologies impact societal factors sometimes with negative environmental consequences. Only a few PBP environmental studies have conducted a sLCA. The value of such an assessment along with a TEA, LCC, and eLCA may spotlight where and how different technologies impact human sustainable development (for example, improving living conditions by providing appropriately salaried positions and safe employee working conditions) (101). An additional benefit to screening technology alternatives eases employees to safely execute their jobs or identify alternatives less environmentally harsh. The challenge with a sLCA is identifying meaningful factors or boundaries providing significant data. Often factors are unique to a location being analyzed. This is especially challenging in developing countries where data are not generated because technology is not deployed in these areas. Forensic investigators often analyze case studies after an accident or tragedy occurs in a production environment. This type of analysis will identify addressable hot spots to be rectified. Finally, several factors previously used to examine softer data variables like personnel turnover, organizational trust, fair employment practices, gender equality, diversity, and other similar items can improve environmental impact and sustainability by recognizing and rewarding engaged employees consciously doing their jobs. Furthermore, it ultimately impacts protein quality and selling price. Which variables are important and should be codified will make this analysis more meaningful and enable additional studies on PBP raw materials, processes used to manufacture PBPC and PBPI, protein-containing food formulations, and ultimately stakeholder acceptance (51). Ultimate stakeholders are consumers, but researchers, policymakers, and regulators also benefit from data as they establish future guidelines in relation to health benefits and environmental impacts.

A functional unit (FU) is a designated comparison set point to relate environmental data. For example, most researchers will set a PBP FU as per kilogram protein manufactured or per a given PBP quantity in a food serving and use environmental data generated from the FU to compare against identical FU of an animal-based protein (57). This concept is extended to contrast environmental impact of switching from traditional animal-based to a PBP. Recently, it is proposed to further include other important nutrients as FU in Nutritional Life Cycle Assessments (nLCA). Nutritionally unified FU such as 100 g protein or other nutrient indices such as minerals, fiber, starch, or other micronutrients can have environmental impacts (58). Unfortunately, these data are totally dependent upon circumstances for which it was analyzed, and any differences in a manufacturing process make it difficult to compare one PBP to another of the same variety (59). This area is rapidly evolving, and researchers will further delineate future analysis parameters.

Many factors directly or indirectly contribute to challenges of commercial PBPC and PBPI offering at cost parity with ADP. This issue originates with farmer/producer actors in the supply chain but probably weaves through every supply chain phase culminating when consumers purchase products containing PBP. Fertilizer and fuel costs since 2021 have significantly impacted farm gate proceeds. PBP manufacturing companies have maintained higher margins on PBPC and PBPI products sold to food formulators, as they establish supply chains and recover capital costs for new manufacturing plants. Costs in recent months are stabilizing and, in some sectors, undergoing deflation because larger PBP manufacturers and food formulators are reaching manufacturing scales to reduce operating costs. Conventional meat prices have also increased, and cost differences are narrowing. PBMA and PBDA product categories have struggled after robust launches and market gains during the pandemic, while other categories using PBP in their formulas, like nutrition bars and beverages, have maintained strong market strength. The PBP industry and finished food product companies believe that the industry is seeing a momentary negative blip in consumer interest. Marketers of these products are also exploiting emerging environmental assessment data to their advantage with climate conscious consumers. The industry’s dedication to continuous improvement in these categories from PBP discovery to new protein foods is needed for food security as year 2050 approaches.

Finally, location consideration should be given on where to build future PBP manufacturing facilities. Future protein supply will be needed in countries rapidly increasing their protein consumption. Even in India where the perception not much meat is consumed, over 65% of the population of 1.4 billion does and their consumption is increasing. A multinational concerted effort is required to educate consumers to consume animal products in moderation for health and environmental preservation and to include PBP in daily dietary planning. These geographical considerations also warrant and will inform further research on development and optimization of distributed manufacturing strategies and robust supply chains for different PBP products.

## Conclusion

Total livestock agriculture elimination is not necessary to mitigate climate change and from a global financial perspective would have profound effects on dozens of adjacent industries. The livestock agriculture industry is learning more about GHGe with research data and improved assessment models. Better animal feeding programs and animal rearing practices are making an environmental difference (102). In fact, aerosol propellants are under intense scrutiny as being more responsible for climate warming than GHGe (103). A better option would be to expend our energy (pun intended) to focus on promoting flexitarian diets whereby the global population reduces their animal-based products consumption by 25% through increased dietary PBP use. In addition to being environmentally better and healthier for the consumer, global economic impact from those actions would be less than building or improving facilities to be more environmentally friendly or eradicating adjacent industries. For the sake of our planet, as a society, we must curb our animal-based protein consumption and accept PBP.

To make progress in preserving our planet for future mankind, the agri-food ecosystem must be viewed as part of the solution rather than only being discussed as part of the problem. We in professional ranks addressing these issues should not be so naive as to believe technology will fix all existing problems (and those we do not know about yet) while ignoring societal needs and socioeconomic conditions to support new technologies (104). It will be important to include community-led and government-supported stakeholders in this transformation for ultimate success. Adequate financing and investment must be solicited from diversified sources to adequately address transformation of our food system toward a more equitable, sustainable, and resilient system preserving food security and planet Earth for centuries to come. Several other factors must be integrated into our thinking including equity, ethics, safety, and security. We will not quickly satisfy critics, but our efforts must work toward consensus and collaborative action.

## Data Availability

There are no data underlying this work.
